# Supplementary material for: Left-handed metamaterial bandpass filter for GPS, Earth Exploration-Satellite and WiMAX frequency sensing applications
Source: PLoS One. 2019 Nov 12;14(11):e0224478. doi: 10.1371/journal.pone.0224478 (PMC6850711; doi:10.1371/journal.pone.0224478)
Supplement: S1 File — (PDF) [file pone.0224478.s001.pdf]

Design methodology

The process flow starts with an extensive literature review on the bandpass filter and its particular applications from where the requirements of filter are collected according to the specifications. Once the filter's geometrical parameters and dielectric substrate are specified, the filter models are ready for simulation. Meanwhile the familiarization with the simulation software CST microwave studio simulator has been performed. Next, the design is simulated, and its performance has been inspected until the point that an acceptable outcome is acquired. The next step is the fabrication where the prototype will be made. Eventually, the performances of the prototype are measured in Vector network analyzer to validate the simulation and to verify the technical specifications. The steps of the proposed methodology are described in detail in following subsections.

Design Specifications:

The beginning of a filter design process is the gathering of targeted specifications according to the application of the filter and review process of the literature. Initially the characteristics of metamaterials for filter application have been reviewed that includes permittivity, permeability and refractive index to set a detail for outlining. In the meantime, acquaintance the simulation software has been completed. Subsequently, in view of the metamaterials and legitimate model, the filters are designed. The process of fabrication and measurement are performed after finishing the design and numerical investigation. According to the literature review, the specifications for the filter design are listed in the Table-below

Table Design specifications for the proposed filters

Filter Characteristics	Bandpass filter with multiple parasitic strips	Bandpass filter with dual parasitic strips
Operation band	L and S-band (1-4GHz)	L and S-band (1-4GHz)
Metamaterial Type	ENG	ENG
Layer	Single	Single
Input Impedance	50 Ω	50 Ω
Profile	Small, Compact and Planar	Small, Compact and Planar

Weight	Light	Light
Shape	Rectangular	Rectangular

The design, fabrication and measurement procedure can be divided in the following stages:

Stage 1: Metamaterial (Resonator) Design

- Selection of electromagnetic computing tool
- Construction of metamaterial using CST
- Selection of boundary condition
- Run the simulation for desired frequency
- Observation on Scattering parameters

Stage 2: Characterization of the metamaterial

- Evaluation of the effective medium ratio of metamaterial
- Selection of proper characterization method
- Calculation of the effective parameters
- Characterization of metamaterial

Stage 3: Metamaterial based Filter design

- Selection of the technique
- Filter design using metamaterial
- Simulation in EM simulator for the desired frequency range
- Examine performances
- Recording of transmission properties from the simulations

Stage 4: Fabrication and Measurement

- Fabrication of the prototype using LPKF S63 PCB prototyping machine and PCB cutter
- Selection of measurement method
- S-parameters measurement of the prototype
- Comparison of measured results with the simulated results
