# Supplementary material for: Left-handed metamaterial bandpass filter for GPS, Earth Exploration-Satellite and WiMAX frequency sensing applications
Source: PLoS One. 2019 Nov 12;14(11):e0224478. doi: 10.1371/journal.pone.0224478 (PMC6850711; doi:10.1371/journal.pone.0224478)
Supplement: Attachment — Submitted filename: Answer to the Reviewers Comments [PONE-D-19-12221].docx [file pone.0224478.s002.docx]

**Dear Editor and Reviewers,**

The authors would like to thank the anonymous reviewers and the editor for their effort in reviewing the manuscript and for their valuable and constructive comments and fruitful observations that helped in improving the quality of the manuscript to a publishable standard. Detailed below are some responses to the reviewers’ comments and suggestions. Reviewers’ questions and comments are in BLACK and the author’s answers and comments are in **BLUE**. Also, the correction in the revised manuscript marked in **RED**.

**Reviewer’s Comments 1:1**

The quality of some figures is not very high, such as Fig.3 and Fig. 5, which need to be revised.

**Author’s Response 1:1**

Thank you for the comment. The quality of the figures has been improved and the updated figures are included in the revised manuscript, which is marked in red.

**Reviewer’s Comments 1:2**

In Fig. 6, what is the effect of the parasitic strips on two sides? Did the authors simulate the results with these strips and without them for comparisons? If the simulated results between them are the same, it is meaningless with them.

**Author’s Response 1:2**

Thank you for the comment. There is significant effect of the parasitic strips to the structure. The use of parasitic strips increases the overall inductance to the circuit, and as a result, the resonant frequencies shifted to the lower frequencies. The difference can be found in Figure 8 (a) and 8 (b). Moreover, the introduction of the parasitic strips induces more surface current to the entire circuit than without parasitic structure made it more feasible for the filter operation (can be found in Figure 9).

**Reviewer’s Comments 1:3**

The previously reported works for references to address the recent research status are not enough. Some recent works are suggested to be added for better understanding this work, such as

[1] “Miniaturized notch-band UWB bandpass filters using interdigital-coupled feed-line structure,” Microwave and Optical Technology Letters, vol. 56, no. 10, pp. 2215-2217, Oct. 2014.

[2] “Planar dual- and tri-band bandpass filters using single improved ring resonator and simple feed scheme,” Microwave and Optical Technology Letters, vol. 56, no. 3, pp. 574-577, March. 2014.

[3] “High-selectivity wideband bandpass filter using simple coupled lines with multiple transmission poles and zeros,” IEEE Microw. Wireless Compon. Lett., vol. 29, no. 2, pp. 107-109, 2019.

[4] “High-selectivity bandpass filter using six pairs of quarter-wavelength coupled lines,” Electronics Letters, vol. 55, no. 9, pp. 544-546, 2019.

**It is better if the authors tabulate a table for performance comparisons.**

**Author’s Response 1:3**

Thank you for your suggestion. All the recommended articles and criticism are included in the revised manuscript, as well cited the manuscript, which is marked in red.

Moreover, a performance comparison with other similar work has been included in the revised manuscript in Table 1, which is marked in red. In addition, the comparison table 3 and related discussion is shown below:

TABLE 3	Comparison of the proposed bandpass filters with previous works

| Previous works | Speciallity | Size (mm) | Opt./Cent. Freq.(GHz) | Application |
| --- | --- | --- | --- | --- |
| Xu et al. 2018 | Dual mode resonators with multiple Stubs | 79 | 2.1 | High-selectivity bandpass filters |
| Gorur et al. 2014 | Metamaterial based filter | 27 | 2.45, 3.5 | WLAN and Wimax |
| Athukorala et al. 2018 | Dual mode open loop  microstrip resonators | 40 | 0–4 | Controllable transmission zeros |
| Hong et al. 2007 | Four pole with dual mode resonators | 34 | 0.5–2.5 | Dual mode microstrip filter |
| Hua et al. 2011 | Dual mode stub-loaded  resonator | 54 | 0–4.5 | Tunable notch |
| Proposed Filters | Triple split ring resonators and stub-centered triple split ring resonator | 30 | 1.55, 2.70, 3.65 and 1.51, 2.45, 3.65 | GPS, IOT, WLAN and WiMAX |

Xu et al. (2018) proposed a dual mode resonator based high-selectivity bandpass filter, where the centre frequency of the filter is 2.1 GHz. The filter operates in S-band but the length of structure is 79 mm. Gorur et al. (2014) also presented a metamaterial based 27 mm bandpass filter with centre frequencies at 2.45 and 3.5 GHz respectively. Though the filter is suitable for dual band operation, but still it does not have dual mode and does not support GPS or IOT application. Athukoral et al. (2018) and Hua et al. (2011) worked on dual mode resonators separately. Both the filters operate in L and S-band frequencies, but the size of the filters are higher than the proposed filters (more than 30 mm). However, the filters are applicable for controllable transmission zeros and tunable transmission notch respectively. Hong et al. (2007) proposed a dual mode resonator based four pole bandpass filter with operating frequencies 0.5 to 2.5 GHz. The filter operates well for dual mode microstrip filter, but it cannot be used multi band applications. All the bandpass filters mentioned above are highly effective in their particular applications, but neither of them covers more than two bands like the proposed filter does. Moreover, the proposed filter in the combination of metamaterial based dual mode resonators with double negative characteristics at the filtering frequencies, which is absent in the above-mentioned works. Hence, the smaller size of the filter and metamaterial characteristics made it unique from the other bandpass filters.

**Reviewer’s Comments 1:4**

The sign symbol of Return loss and Insertion loss is plus rather than minus, which is the most basic concept in microwave engineering. Therefore, the expression of Fig. 8 is incorrect. Please revise it.

**Author’s Response 1:4**

Thank you for your valuable comment. According to the reviewer’s comments the Figure 8 has been modified, which is marked in red in the revised manuscript.

**Reviewer’s Comments 2:1**

A detailed design procedure for a given specification should be provided for the filter design.

**Author’s Response 2:1**

Thank you for the good query. According to the reviewer’s comments, the design and fabrication process of a bandpass filter is described below and all information is included in the revised manuscript, as a supplementary file.

The process flow starts with an extensive literature review on the bandpass filter and its particular applications from where the requirements of filter are collected according to the specifications. Once the filter’s geometrical parameters and dielectric substrate are specified, the filter models are ready for simulation. Meanwhile the familiarization with the simulation software CST microwave studio simulator has been performed. Next, the design is simulated, and its performance has been inspected until the point that an acceptable outcome is acquired. The next step is the fabrication where the prototype will be made. Eventually, the performances of the prototype are measured in Vector network analyzer to validate the simulation and to verify the technical specifications. The steps of the proposed methodology are described in detail in following subsections.

**Design Specifications:**

The beginning of a filter design process is the gathering of targeted specifications according to the application of the filter and review process of the literature. Initially the characteristics of metamaterials for filter application have been reviewed that includes permittivity, permeability and refractive index to set a detail for outlining. In the meantime, acquaintance the simulation software has been completed. Subsequently, in view of the metamaterials and legitimate model, the filters are designed. The process of fabrication and measurement are performed after finishing the design and numerical investigation. According to the literature review, the specifications for the filter design are listed in the Table-3.1.

Table 3.1	Design specifications for the proposed filters

| Filter Characteristics | Bandpass filter with multiple parasitic strips | Bandpass filter with dual parasitic strips |
| --- | --- | --- |
| Operation band | L and S-band (1-4GHz) | L and S-band (1-4GHz) |
| Metamaterial Type | ENG | ENG |
| Layer | Single | Single |
| Input Impedance | 50 Ω | 50 Ω |
| Profile | Small, Compact and Planar | Small, Compact and Planar |
| Weight | Light | Light |
| Shape | Rectangular | Rectangular |

The design, fabrication and measurement procedure can be divided in the following stages:

Stage 1: Metamaterial (Resonator) Design

- Selection of electromagnetic computing tool
- Construction of metamaterial using CST
- Selection of boundary condition
- Run the simulation for desired frequency
- Observation on Scattering parameters

Stage 2: Characterization of the metamaterial

- Evaluation of the effective medium ratio of metamaterial
- Selection of proper characterization method
- Calculation of the effective parameters
- Characterization of metamaterial

Stage 3: Metamaterial based Filter design

- Selection of the technique
- Filter design using metamaterial
- Simulation in EM simulator for the desired frequency range
- Examine performances
- Recording of transmission properties from the simulations

Stage 4: Fabrication and Measurement

- Fabrication of the prototype using LPKF S63 PCB prototyping machine and PCB cutter
- Selection of measurement method
- S-parameters measurement of the prototype
- Comparison of measured results with the simulated results

**Reviewer’s Comments 2:2**

Please add a performance comparison with other similar works for displaying the advantages of this work.

**Author’s Response 2:2**

Thank you for your suggestion. A performance comparison with other similar work has been included in the revised manuscript in Table 3, which is marked in red. In addition, the comparison table 3 and related discussion is shown below:

TABLE 3	Comparison of the proposed bandpass filters with previous works

| Previous works | Speciallity | Size (mm) | Opt./Cent. Freq.(GHz) | Application |
| --- | --- | --- | --- | --- |
| Xu et al. 2018 | Dual mode resonators with multiple Stubs | 79 | 2.1 | High-selectivity bandpass filters |
| Gorur et al. 2014 | Metamaterial based filter | 27 | 2.45, 3.5 | WLAN and Wimax |
| Athukorala et al. 2018 | Dual mode open loop  microstrip resonators | 40 | 0–4 | Controllable transmission zeros |
| Hong et al. 2007 | Four pole with dual mode resonators | 34 | 0.5–2.5 | Dual mode microstrip filter |
| Hua et al. 2011 | Dual mode stub-loaded  resonator | 54 | 0–4.5 | Tunable notch |
| Proposed Filters | Triple split ring resonators and stub-centered triple split ring resonator | 30 | 1.55, 2.70, 3.65 and 1.51, 2.45, 3.65 | GPS, IOT, WLAN and WiMAX |

Xu et al. (2018) proposed a dual mode resonator based high-selectivity bandpass filter, where the centre frequency of the filter is 2.1 GHz. The filter operates in S-band but the length of structure is 79 mm. Gorur et al. (2014) also presented a metamaterial based 27 mm bandpass filter with centre frequencies at 2.45 and 3.5 GHz respectively. Though the filter is suitable for dual band operation, but still it does not have dual mode and does not support GPS or IOT application. Athukoral et al. (2018) and Hua et al. (2011) worked on dual mode resonators separately. Both the filters operate in L and S-band frequencies, but the size of the filters are higher than the proposed filters (more than 30 mm). However, the filters are applicable for controllable transmission zeros and tunable transmission notch respectively. Hong et al. (2007) proposed a dual mode resonator based four pole bandpass filter with operating frequencies 0.5 to 2.5 GHz. The filter operates well for dual mode microstrip filter, but it cannot be used multi band applications. All the bandpass filters mentioned above are highly effective in their particular applications, but neither of them covers more than two bands like the proposed filter does. Moreover, the proposed filter in the combination of metamaterial based dual mode resonators with double negative characteristics at the filtering frequencies, which is absent in the above-mentioned works. Hence, the smaller size of the filter and metamaterial characteristics made it unique from the other bandpass filters.

**Reviewer’s Comments 2:3**

The circuit size should be given.

**Author’s Response 2:3**

According to the strip line theory [1], capacitance is produced in the strip line gap and inductance is created in metal strip. Based on the theory strip line and gap are converted into equivalent circuit. Hence, the inductance of the strip line is equal to

*L(nH) = 2×10^-4^ l [ln* $(\frac{l}{w+t})$ *+ 1.193 + 0.2235* $(\frac{w+t}{l})$*]*	(1)

The magnitude of L_1_, L_2_, L_3_, L_4_, L_4_, L_5_, L_6_, L_7_, L_8_, L_9_ and L_10_ in the equivalent circuit calculated using equation (1).

The capacitances of the metal strip line and SRR gap are determined as follows [1]:

*C(pF) =* $\varepsilon_{0}\varepsilon_{e}F(k)$	(2)

$\varepsilon_{e}=1+ \frac{\left( \varepsilon_{r}-1 \right)F(k)}{F(k1)}$	(3)

$k1= \frac{\sinh\frac{\pi a}{2h}}{\sinh\frac{\pi a}{2h}}$	(4)

$F\left( k \right)=\left\{ \begin{aligned} \frac{1}{\pi} \ln\left( 2\frac{1+ \sqrt{k}}{1- \sqrt{k}} \right) & for 0.707<k\leq1 \\ \pi\ln\left( 2\frac{1+ \sqrt{k'}}{1- \sqrt{k'}} \right)^{-1} & for 0<k\leq0.707 \end{aligned} \right.$	(5)

where $\varepsilon_{0}$ is the free space permittivity, $\varepsilon_{e}$ is the effective dielectric constant of the microstrip line with width W,$\varepsilon_{r}$ is the relative permittivity of the substrate, $F\left( k \right)$ is the ratio between the complete elliptic integral of the first solution $K(k)$ and its complement $K'(k)$, and h is the thickness of the substrate.

Reference:

Bahl I, Bhartia P. Microwave solid state circuit design. 2nd ed. Jhon Wiley & Sons, Inc., Hoboken, New Jersey, 2003, pp-27- 72. ISBN 0-1471-20755-1.

**Reviewer’s Comments 3:1**

The filter analysis is given in this manuscript, but filter synthesis is not provided. Therefore, it is difficult to follow the proposed work when the design specifications are different. At least, a detailed design procedure should be given for more general cases.

**Author’s Response 3:1**

Thank you for the good query. According to the reviewer’s comments, the design and fabrication process of a bandpass filter is described below and all information is included in the revised manuscript, as a supplementary file.

The design, fabrication and measurement procedure can be divided in the following stages:

Stage 1: Metamaterial (Resonator) Design

- Selection of electromagnetic computing tool
- Construction of metamaterial using CST
- Selection of boundary condition
- Run the simulation for desired frequency
- Observation on Scattering parameters

Stage 2: Characterization of the metamaterial

- Evaluation of the effective medium ratio of metamaterial
- Selection of proper characterization method
- Calculation of the effective parameters
- Characterization of metamaterial

Stage 3: Metamaterial based Filter design

- Selection of the technique
- Filter design using metamaterial
- Simulation in EM simulator for the desired frequency range
- Examine performances
- Recording of transmission properties from the simulations

Stage 4: Fabrication and Measurement

- Fabrication of the prototype using LPKF S63 PCB prototyping machine and PCB cutter
- Selection of measurement method
- S-parameters measurement of the prototype
- Comparison of measured results with the simulated results

**Reviewer’s Comments 3:2**

For the triple-band filter, it is better to add the simulated and measured group delay for the passband.

**Author’s Response 3:2**

According to the reviewer’s comments the group delay results have been included in the revised manuscript, which is marked in red.

Group delay is defined as the rate of change of transmission phase angle with respect to frequency. In filter, group delay is very important to show the bandpass accuracy i.e. signal is passing without any dispersion or loss. The group delay of a filter is nearly proportional to its order. On the other hand, filter group delay is inversely proportional to filter bandwidth i.e. small percentage bandwidth filters have large group delay. In our developed filter, we found resonance frequency at 1.55, 2.70 and 3.60 GHz respectively, whereas we found group delay pick at our desired frequency in simulation, which proves that the performance of bandpass filter is enough good that it can pass the desired frequency with less noise and less loss. However, measured results followed the simulated group delay shown despite of some minor detachment. This minor difference usually happens due to some possible reasons like

1. There may be a calibration error associated with the Agilent N5227 vector network analyzer by Agilent N4694-60001 Ecal.
2. Impedance mismatch between the SMA connectors and the feedline.
3. Minor fabrication error.
4. Some insertion loss due to minor soldering problems of SMA connectors.
5. Using the Agilent N5227 vector network analyzer in the room, there might be some reflective metallic components for what some reflection exits in the measurement which caused the variation of the results.

Figure11: Simulated and measured group delay for the passband filter.

**Reviewer’s Comments 3:3**

The pictures in this paper are not very clear, especially for Fig. 11.

**Author’s Response 3:3**

Thank you for your comment. According to the reviewer’s suggestion, the quality of the pictures in this paper has been improved, which is shown in the revised manuscript Fig. 12.

**Reviewer’s Comments 3:4**

It is better to give the circuit size of the fabricated filter.

**Author’s Response 3:4**

According to the strip line theory [1], capacitance is produced in the strip line gap and inductance is created in metal strip. Based on the theory strip line and gap are converted into equivalent circuit. Hence, the inductance of the strip line is equal to

*L(nH) = 2×10^-4^ l [ln* $(\frac{l}{w+t})$ *+ 1.193 + 0.2235* $(\frac{w+t}{l})$*]*	(1)

The magnitude of L_1_, L_2_, L_3_, L_4_, L_4_, L_5_, L_6_, L_7_, L_8_, L_9_ and L_10_ in the equivalent circuit calculated using equation (1).

The capacitances of the metal strip line and SRR gap are determined as follows [1]:

*C(pF) =* $\varepsilon_{0}\varepsilon_{e}F(k)$	(2)

$\varepsilon_{e}=1+ \frac{\left( \varepsilon_{r}-1 \right)F(k)}{F(k1)}$	(3)

$k1= \frac{\sinh\frac{\pi a}{2h}}{\sinh\frac{\pi a}{2h}}$	(4)

$F\left( k \right)=\left\{ \begin{aligned} \frac{1}{\pi} \ln\left( 2\frac{1+ \sqrt{k}}{1- \sqrt{k}} \right) & for 0.707<k\leq1 \\ \pi\ln\left( 2\frac{1+ \sqrt{k'}}{1- \sqrt{k'}} \right)^{-1} & for 0<k\leq0.707 \end{aligned} \right.$	(5)

where $\varepsilon_{0}$ is the free space permittivity, $\varepsilon_{e}$ is the effective dielectric constant of the microstrip line with width W,$\varepsilon_{r}$ is the relative permittivity of the substrate, $F\left( k \right)$ is the ratio between the complete elliptic integral of the first solution $K(k)$ and its complement $K'(k)$, and h is the thickness of the substrate.

Reference:

Bahl I, Bhartia P. Microwave solid state circuit design. 2nd ed. Jhon Wiley & Sons, Inc., Hoboken, New Jersey, 2003, pp-27- 72. ISBN 0-1471-20755-1.

**Reviewer’s Comments 4:1**

Provide the step from equation (6) to equation (7).

**Author’s Response 4:1**

For even-mode excitation, there is no current flow through the symmetrical plane. When we ignore the discontinuity of the folded section, the input admittance for the even mode can be expressed as [23]:

$Y_{ie}=jY_{1}\frac{\left( 2Y_{1}Y_{e}+Y_{3}Y_{e}+2{Y^{2}}_{e} \right)tan\theta-Y_{1}Y_{3}{tan}^{3}\theta}{2Y_{1}Y_{e}-(Y_{1}Y_{3}+Y_{3}Y_{e}+2{Y^{2}}_{e}){tan}^{2}\theta}$	(6)

From the even-mode resonance condition of Yie = 0, we obtain the following equation,

${tan}^{2}\theta=K_{2}=\frac{Y_{e}}{Y_{1}}+\frac{2{Y^{2}}_{e}}{Y_{1}Y_{3}}+\frac{{2Y}_{e}}{Y_{3}}$	(7)

**Reviewer’s Comments 4:2**

The author design unit cell for 3.11GHz frequency. But the filter is designed for 1.55GHz, 2.7GHz, and 3.6GHz operating frequency by employing the unit cell. Give a proper explanation of it.

**Author’s Response 4:2**

The objective of this research was to design on open loop resonator-based band pass filter for lower frequencies (especially less than 4GHz). In this regard, an extensive literature review has been done and got the concept of implementing metamaterials into the microstrip line. However, the metamaterials do occur in periodic structure. So, two separate unit cells are established to attain the higher frequencies. Then the authors wanted to make the filter unique and wanted to apply the filter to more than one operating frequencies. In this regard, the size of the substrate was increased and filled the vacant spaces with metal strips to shift the resonances to the lower frequencies (applying metal strips increased the total inductance in the circuit).

**Reviewer’s Comments 4:3**

In the introduction section, the author gives more emphasis on the metamaterial literature survey and not for the filter. Write more literature about the filter.

**Author’s Response 4:3**

Thank you for your comment. The authors have gone through the revised manuscript and added more literature about the filter, which is marked in red.

**Reviewer’s Comments 4:4**

Remove some part of the basic of the metamaterial.

**Author’s Response 4:4**

Thank you for the suggestion. The authors have removed some past of the basic of the metamaterial in the revised manuscript.

**Reviewer’s Comments 4:5**

There are too many references for Alam .M and Islam .M work.

**Author’s Response 4:5**

Author checked the manuscript’s reference section and update some new references in the revised manuscript.

**Reviewer’s Comments 4:6**

What is OLR?

**Author’s Response 4:6**

OLR is Open loop resonator.

**Reviewer’s Comments 4:7**

Maintain the representation of T or T.

**Author’s Response 4:7**

All the scientific notations are corrected in the revised manuscript.

**Reviewer’s Comments 4:8**

Write fig. 4(b) as Fig. 4(b)

**Author’s Response 4:8**

Corrected in revised manuscript.

**Reviewer’s Comments 4:9**

Write Table2 notation at the right place.

**Author’s Response 4:9**

Table 2 notation is corrected in revised manuscript.

**Reviewer’s Comments 4:10**

Fig. 2 descriptive caption is written in two places.

**Author’s Response 4:10**

One descriptive caption is removed in revised manuscript.

**Reviewer’s Comments 4:11**

Maintain the figure descriptive caption style same throughout the manuscript.

**Author’s Response 4:11**

Based on the reviewer suggestion figure caption styles are changed throughout the manuscript.

**Reviewer’s Comments 5:1**

The proposed structure is no novel.

**Author’s Response 5:1**

The concept of making this filter structure is to make a fusion between metamaterial and microstrip structure. In this regard, the authors have designed unique rectangular metamaterial structure within the range of 1 to 4 GHz. Then the authors have decided to combine the metamaterial structure with microstrip line. But the overall structure should have followed the mode (even and odd) of the filter concept. However, the authors have claimed the overall structure to metamaterial as well. They have shown the metamaterial characteristics for the filter structure as well. We all know that metamaterial structure has to be in array formation. Therefore, a 1*2 array has been formatted on the microstrip line. The fusion of unique metamaterial design with microstrip structure makes it different from other structures. Finally, the authors concerned that no body used this type of metamaterials design for filter application.

**Reviewer’s Comments 5:2**

The performance of the proposed filter is not good. The selectivity should be improved further.

**Author’s Response 5:2**

Thank you for this valuable suggestion. According to the suggestion, the authors tried to improve the selectivity of the bandpass filter in some possible ways. However, the authors measured the group delay of the proposed bandpass filter to check the performance at the pass band. From the results, the authors could say that performance of bandpass filter is enough good that it can pass the desired frequency [1.55GHz, 2.70GHz, 3.6GHz] with less noise and less loss. The measured results followed the simulated group delay shown despite of some minor detachment.

**Reviewer’s Comments 5:3**

The author should compare the performance of the proposed filter with some recently published ones

**Author’s Response 5:3**

Thank you for your suggestion. A performance comparison with other similar work has been included in the revised manuscript in Table 3, which is marked in red. In addition, the comparison table 3 and related discussion is shown below:

TABLE 3	Comparison of the proposed bandpass filters with previous works

| Previous works | Speciallity | Size (mm) | Opt./Cent. Freq.(GHz) | Application |
| --- | --- | --- | --- | --- |
| Xu et al. 2018 | Dual mode resonators with multiple Stubs | 79 | 2.1 | High-selectivity bandpass filters |
| Gorur et al. 2014 | Metamaterial based filter | 27 | 2.45, 3.5 | WLAN and Wimax |
| Athukorala et al. 2018 | Dual mode open loop  microstrip resonators | 40 | 0–4 | Controllable transmission zeros |
| Hong et al. 2007 | Four pole with dual mode resonators | 34 | 0.5–2.5 | Dual mode microstrip filter |
| Hua et al. 2011 | Dual mode stub-loaded  resonator | 54 | 0–4.5 | Tunable notch |
| Proposed Filters | Triple split ring resonators and stub-centered triple split ring resonator | 30 | 1.55, 2.70, 3.65 and 1.51, 2.45, 3.65 | GPS, IOT, WLAN and WiMAX |

Xu et al. (2018) proposed a dual mode resonator based high-selectivity bandpass filter, where the centre frequency of the filter is 2.1 GHz. The filter operates in S-band but the length of structure is 79 mm. Gorur et al. (2014) also presented a metamaterial based 27 mm bandpass filter with centre frequencies at 2.45 and 3.5 GHz respectively. Though the filter is suitable for dual band operation, but still it does not have dual mode and does not support GPS or IOT application. Athukoral et al. (2018) and Hua et al. (2011) worked on dual mode resonators separately. Both the filters operate in L and S-band frequencies, but the size of the filters are higher than the proposed filters (more than 30 mm). However, the filters are applicable for controllable transmission zeros and tunable transmission notch respectively. Hong et al. (2007) proposed a dual mode resonator based four pole bandpass filter with operating frequencies 0.5 to 2.5 GHz. The filter operates well for dual mode microstrip filter but it cannot be used multi band applications. All the bandpass filters mentioned above are highly effective in their particular applications, but neither of them cover more than two bands like the proposed filter does. Moreover, the proposed filter in the combination of metamaterial based dual mode resonators with double negative characteristics at the filtering frequencies, which is absent in the above mentioned works. Hence, the smaller size of the filter and metamaterial characteristics made it unique from the other bandpass filters.

I look forward to give further clarification if required, and your favorable consideration on the above matter is greatly appreciated.

Sincerely yours

===========================

Md. Jubaer Alam

Corresponding Author
